# Transparent Electrodes Based on Crack-Templated Metallic Networks for Next-Generation Optoelectronics

**DOI:** 10.3390/ma18133091

**Published:** 2025-06-30

**Authors:** Eleonora Sofia Cama, Mariacecilia Pasini, Francesco Galeotti, Umberto Giovanella

**Affiliations:** National Research Council (CNR), Institute of Chemical Sciences and Technologies (SCITEC), Via Alfonso Corti 12, 20133 Milan, Italy; eleonora.cama@scitec.cnr.it (E.S.C.); mariacecilia.pasini@scitec.cnr.it (M.P.); francesco.galeotti@scitec.cnr.it (F.G.)

**Keywords:** transparent conductive electrode, flexible electronics, self-cracking, crack lithography, metal mesh, physical vapor deposition

## Abstract

Transparent conductive electrodes (TCEs) are essential components in modern optoelectronic devices, including organic light-emitting diodes and solar cells, sensors, and flexible displays. Indium tin oxide has been the dominant material for TCEs due to its high transparency and conductivity. However, its brittleness, high cost, and increasingly limited availability pose significant challenges for electronics. Crack-template (CT)-assisted fabrication has emerged as a promising technique to develop metal mesh-based TCEs with superior mechanical flexibility, high conductivity, and excellent optical transmittance. This technique leverages the spontaneous formation of random and continuous microcrack networks in sacrificial templates, followed by metal deposition (e.g., Cu, Ag, Al, etc.), to produce highly conductive, scalable, and low-cost electrodes. Various crack formation strategies, including controlled drying of polymer suspensions, mechanical strain engineering, and thermal processing, have been explored to tailor electrode properties. Recent studies have demonstrated that crack-templated TCEs can achieve transmittance values exceeding 85% and sheet resistances below 10 Ω/sq, with mesh line widths as low as ~40 nm. Moreover, these electrodes exhibit enhanced stretchability and robustness under mechanical deformation, outperforming ITO in bend and fatigue tests. This review aims to explore recent advancements in CT engineering, highlighting key fabrication methods, performance metrics across different metals and substrates, and presenting examples of its applications in optoelectronic devices. Additionally, it will examine current challenges and future prospects for the widespread adoption of this emerging technology.

## 1. Introduction

Indium tin oxide (ITO) has long been the material of choice for transparent electrodes in optoelectronic devices, such as OLEDs, OSCs, organic light-emitting transistors (OLET), sensors, and touchscreens, due to its excellent optical transparency and electrical conductivity [1,2]. However, ITO faces significant limitations, including brittleness, high production costs, and the limited availability of indium, a rare and expensive metal [3,4]. These challenges have driven the search for alternative materials that can offer improved flexibility, cost-effectiveness, and scalability, while maintaining high performance in terms of both transparency and conductivity. Transparent conductive electrodes (TCEs) are essential components in a wide range of advanced optoelectronic devices, as they provide the critical combination of optical transparency and electrical conductivity [1,5], making them indispensable in technologies like touchscreens, OLEDs, photovoltaic cells, and flexible electronics [6,7,8].

Current TCEs span a variety of materials, each with distinct advantages and limitations. Metal nanowires (NWs) such as silver (AgNWs), copper NWs, and gold NWs, along with core–shell structures, offer high electrical conductivity, though AgNWs in particular suffer from poor adhesion and thermal instability [9,10,11,12,13]. Carbon-based materials like graphene, graphene oxide, and carbon nanotubes (CNTs) are prized for their flexibility and mechanical strength, but often face challenges related to high resistance and difficult processing [14,15]. Metal oxides, including tin oxide (SnO_2_), zinc oxide (ZnO), and indium oxide (In_2_O_3_), are widely used for their transparency and conductivity, often coupled with metallic NWs, but typically require vacuum-based deposition and show brittle structures [16]. Conductive polymers such as PEDOT:PSS, polypyrrole (PPY), and polyaniline (PANI) provide flexibility and ease of processing, though they generally offer lower conductivity [17]. Meanwhile, transition metal dichalcogenides (TMDCs) like MoS_2_ and WS_2_ [18,19], as well as emerging silicene and MXenes [20], exhibit promising optoelectronic properties and tunable work functions (WFs, defined as the minimum energy needed to extract an electron from its surface, usually measured in electron volts (eV) [21]), expanding the landscape of materials for next-generation TCEs [22,23,24].

**Figure 1:**
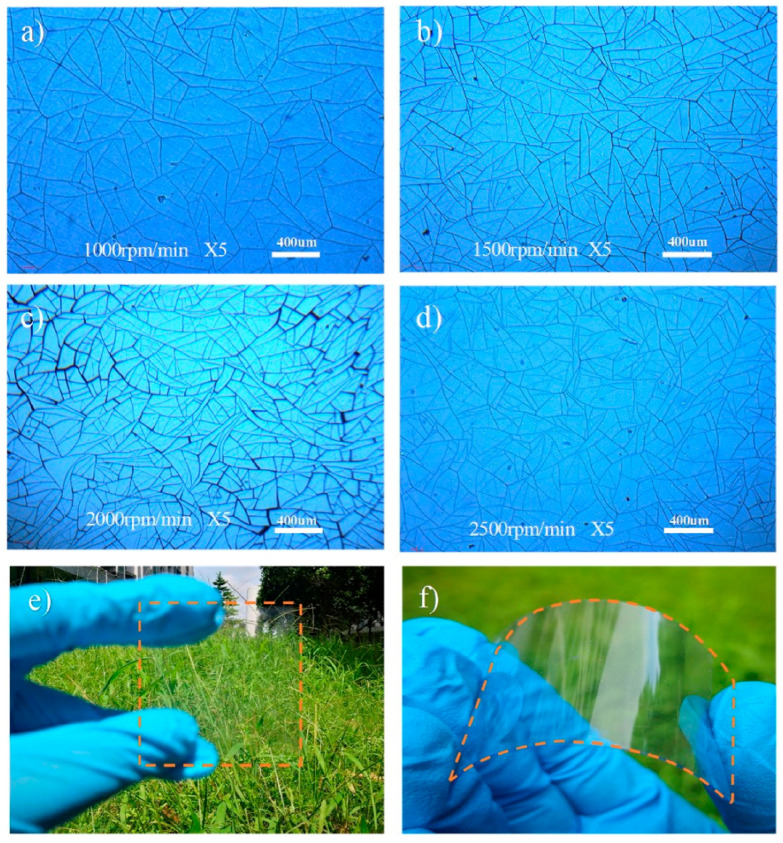
Optical microscope images (5× magnification) of Ag networks for flexible thermochromic device applications, formed from cracked acrylic colloidal dispersion at varying spin coating speeds: (**a**) 1000 rpm, (**b**) 1500 rpm, (**c**) 2000 rpm, and (**d**) 2500 rpm. Photographs in (**e**,**f**) illustrate the transparency and flexibility of the Ag micro-networks on a Polyethylene terephthalate (PET) substrate (30 × 30 mm^2^), with the Ag MNs highlighted by orange outlines. Reproduced with permission from ref. [28].

Traditionally, cracks in coatings and films have been perceived as detrimental defects that compromise mechanical strength and overall performance [25]. However, in recent years, the deliberate induction and precise tuning of cracks have been harnessed as functional templates for various applications, a process commonly referred to as “crack lithography” [26,27]. By controlling crack formation through tailored processing (e.g., spin-coating speed, humidity, temperature), materials with customizable features—such as crack density, width, shape, and orientation—can be engineered to optimize optoelectronic properties for flexible TCE applications (Figure 1) [28]. These crack patterns serve as sacrificial templates, facilitating the formation of metal wire networks through subsequent metal deposition.

The concept of CTs has significantly evolved within the field of fracture mechanics. Among the earliest works in this field, Gorokhov et al. in 1998 developed a mask formation technique using controlled cracking in glassy films on semiconductor substrates, enabling the creation of self-aligned patterns for narrow electrodes via electrolytic deposition [29]. Over a decade later, Xu et al. demonstrated a lithography-free method to fabricate nanochannel arrays on polystyrene (PS) surfaces, allowing precise control over channel depth (20–200 nm) and length (up to tens of millimeters), with applications in nanofluidics such as ion enrichment and current rectification [30]. These pioneering studies laid the foundation for crack templating as a scalable, high-precision alternative to conventional lithography.

**Figure 2:**
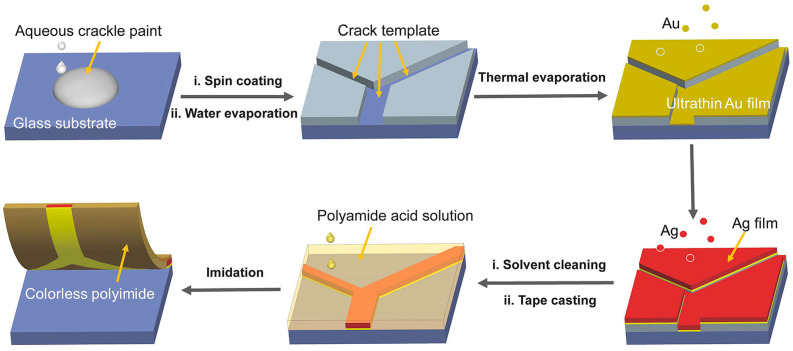
Schematic illustration of one of the possible transfer-printing techniques: fabrication process for the colorless shape memory polyimide embedded with a hybrid (Au/Ag) metal grid. Reproduced with permission from ref. [38].

In contrast to traditional fabrication techniques such as photolithography, electron beam lithography, and nanoimprint lithography, which are often limited by high costs, complex processing steps, and scalability constraints [31,32,33], crack templating provides a cost-effective, scalable, and straightforward alternative for generating conductive networks and channel arrays. Compared to ITO electrodes, which require deposition via sputtering under high-vacuum conditions—necessitating costly instrumentation and limiting the choice of substrates to thermally and mechanically resistant materials such as glass—crack templating offers the significant advantage of being implementable under ambient conditions without the need for specialized equipment. Furthermore, electrodes fabricated via CT approach can be readily transferred onto a broad range of target substrates, including flexible, stretchable, and biodegradable materials, such as PDMS, PET or polyimide (PI) substrates, enabling easier integration into optoelectronic devices, through straightforward transfer-printing techniques (Figure 2) [34,35,36,37,38,39].

One significant advantage of this method is its sustainability. It exploits non-toxic materials like polymers and metals, and mostly operates at lower temperatures, cutting energy consumption. After lift-off, only the metal deposited inside the cracks is retained on the substrate, while the metal deposited on the planar regions is washed away with the removed template. In the study of Rao et al., the recovered metal (Ag) can be reused after template removal, with recovery rates ranging from 76% to 85% of the deposited metal. This recovery process is uniformly applicable to all metals tested, offering a significant sustainability advantage in large-scale fabrication [40]. Additionally, the recycling of residue metallic micro-nanosheets can minimize environmental waste, as demonstrated by Yan et al. through the creation of conductive Ag paste [41].

**Figure 3:**
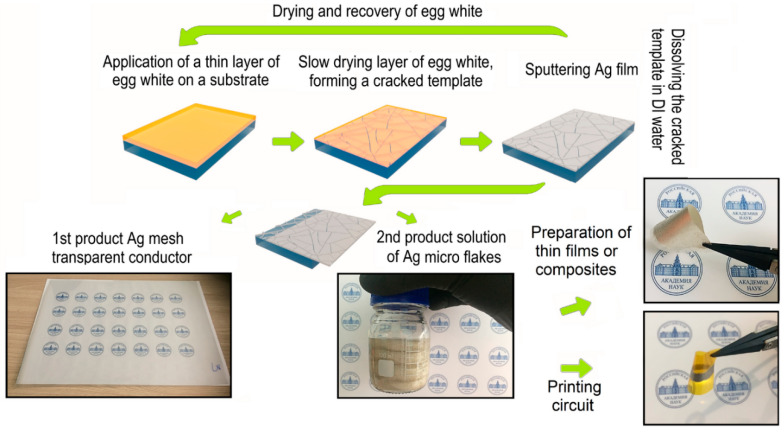
Overview of the fabrication process for producing a silver mesh transparent electrode and silver microflakes. Reproduced with permission from ref. [42].

In another recent work, Voronin and colleagues introduced a waste-free closed cycle for Ag microflakes, purifying them from traces of egg white using water washing and centrifugation, followed by vacuum filtration to form thin films (Figure 3). These Ag microflakes have been used for shielding performance and as a filler in the conductive paste used for the contact pads of an optically transparent heater [42]. This approach promotes sustainability by eliminating harmful chemicals and enhancing the recyclability of materials used in the production of TCEs.

Crack formation in thin films is predominantly governed by the interplay of intrinsic and residual stresses that arise during and after film formation. Intrinsic stress develops in situ due to interactions such as particle–particle forces or particle–substrate adhesion, while residual stress persists after cooling, resulting from structural or thermal changes during processing. When these stresses exceed the material’s fracture strength, cracks initiate, significantly influencing the film’s structural integrity [43].

The fracture mechanisms include solvent evaporation [44], thermal stress induction [45], and mechanical deformation [46], all of which are influenced by material properties (e.g., polymers [47], ceramics [48], and hybrid composites [49]) and processing parameters (e.g., drying rates [50], thermal gradients [51], film thickness [52], and substrate chemistry [53]). Other key factors influencing crack formation include defect accumulation, grain boundaries, and surface irregularities, which act as stress concentrators and crack nucleation sites [54].

An in-depth discussion of the various aspects of the CT technique has been reported in our previous review [43]; in the present work, we specifically focus on the development of TCE for optoelectronic applications.

The discussion integrates recent advancements, challenges, and emerging strategies in this rapidly evolving field, providing an overview of CT applications such as TCE in optoelectronic devices. Owing to their low fabrication cost and the ability to be transferred onto a wide range of target substrates—including flexible and transparent materials—CTs offer a practical and versatile platform for TCE fabrication. Leveraging this technique, researchers can manipulate crack networks to create scalable and versatile ITO-free TCEs for flexible displays, touchscreens, photovoltaics, and wearable electronics.

## 2. Conductive Meshes: Material Selection and Properties

In the design of conductive networks (also indicated as meshes), the choice of material plays a pivotal role in determining the overall electrical, mechanical, and chemical performance of the final structure. Commonly used metals include silver (Ag), copper (Cu), and gold (Au), each offering distinct advantages. Ag has been the most extensively used material for TCEs, mostly in the form of AgNWs, due to its excellent electrical conductivity, optical transparency, and well-established synthesis and processing techniques [55]. Cu has also emerged as a promising candidate for TCEs, offering high electrical conductivity comparable to silver, but at a significantly lower cost and widespread availability [56]. Lastly, Au, although the most expensive, provides excellent chemical stability and corrosion resistance, making it suitable for long-term reliability in harsh environments [57]. The selection among these metals also depends on respective WF and must balance conductivity, stability, cost, and application-specific requirements. Beyond the traditional conductive metals, alternative and hybrid materials are gaining increasing attention for their ability to offer a balanced combination of performance, stability, and cost-efficiency. Aluminum (Al) and tin (Sn) are being investigated as cost-effective alternatives for crack-based conductive meshes in optoelectronic and energy-related applications. In fact, Al meshes demonstrate promising conductivity and scalability for integration in solar cells, while Sn, typically utilized as SnO_2_, is particularly relevant for smart window technologies due to its optical transparency, tunable electrical properties, and compatibility with electrochromic systems [58,59]. Hybrid structures like Au-Ag or Cu-Ag, Ni-Ag composites aim to synergize high conductivity with enhanced mechanical or chemical properties of a secondary metal, improving durability while reducing material costs [38,60]. Metal-oxide hybrids, such as WO_3_/Ag or Al/SnO_2_/WO_3_, further expand functionality by integrating optical transparency, gas sensing capabilities, or electrochromic behavior alongside electrical conductivity [61,62]. Similarly, Vanadium (V)-doped IZO (IZVO) meshes introduce flexibility to indium-based TCEs, making it a promising material for next-generation optoelectronic applications [63].

Table 1 summarizes the main studies on crack-templated metal meshes, highlighting their key performance metrics (such as mesh material, metal deposition technique, and CT material, etc.). The works listed are arranged by material, starting with the most used and ending with mixed/hybrid materials; entries within each category are presented in chronological order. Table 1 also lists the potential applications of CT-TCEs, including cases where a complete device has not yet been demonstrated; the details of the devices that have actually been fabricated are further discussed in Section 3.

### 2.1. Silver

Ag has emerged as the most widely used material in the development of TCEs, initially through the extensive use of AgNWs and more recently as a CT, owing to its excellent electrical conductivity, stability, ease of processing, and compatibility with various substrates and fabrication techniques [71,87].

The thickness of Ag meshes-based CTs typically varies from approximately 40 nm to 10 microns, influenced by factors such as material deposition methods and type of cracking material [34,41]. The control of the thickness enables tailored properties in the templates, which is essential for optimizing their performance in applications like flexible electronics or optoelectronic devices. The variation in thickness affects both the transparency and Rs of the meshes. Typically, silver meshes exhibit transparency around 85% at 550 nm, while their Rs varies from 0.5 to 13 Ω/sq, depending on application to which it is intended [39,71].

Different techniques have been employed for the deposition of Ag in crack-based mesh applications. These include physical vapor deposition (PVD) (e.g., vacuum/thermal evaporation), magnetron sputtering, but also electroplating (EP) and electroless deposition (ED). More recently, Ag ink coating and curing methods have been introduced, offering another versatile approach to fabrication. Each of these techniques provides distinct advantages in terms of control overall mesh properties, with variations in deposition rate, material purity, and equipment complexity and costs.

In the work of Rao et al. [40], Ag deposited via PVD demonstrated superior performance compared to other metals such as Au and Cu. An Ag mesh (AgCP)-TCE deposited on quartz exhibited an average transmittance of 90.5% across a wide spectral range (240 to 3000 nm), with a sheet resistance (Rs) of 2.6 Ω/sq, which is one order of magnitude better than typical 2–3 layer graphene-based TCEs. The haze value of the AgCP-TCE was also notably low, around 2–3%, indicating minimal light scattering. When transferred to a PET substrate, the AgCP-TCE demonstrated a uniform Rs of ~4.7 Ω/sq. Furthermore, AgCP-TCEs/PET featured high flexibility, with minimal changes in Rs (less than 0.5 Ω variation) under repeated bending. The system also demonstrated excellent thermal resistance, with a relatively high value of 420 °C cm^2^/W, making it suitable for flexible transparent heaters.

Suh and co-workers fabricated an Ag-based TCE by first creating a controlled random crack network on a Si_3_N_4_ film deposited on a silicon substrate [34]. AgNWs are then deposited to fill the crack channels using drop-casting and doctor blading, forming bundled AgNW networks. Finally, a UV-curable epoxy resin is applied to the filled template, and a PET film is laminated over it, cured with UV light, and peeled off to transfer the AgNW network onto a flexible PET substrate. The resulting TCE shows high optical transparency (~85%) and low Rs, which is comparable to ITO-based electrodes. It also demonstrates excellent flexibility, with minimal Rs change after over 5000 bending cycles, even under extreme bending conditions (e.g., 50,000 cycles at 3.5 mm bending radius), making it ideal for flexible electronic applications like touch-screen panels.

A combination of deposition techniques can be used to optimize the performance of Ag-based TCEs. For example, in the work of Yang et al. [67], a combination of ED and EP was employed to deposit Ag on glass, with a thicknesses of 100 nm through ED and 2.5 μm through EP. The resulting TCE exhibited high transmittance (80% for ED, 82% for EP) and low Rs (0.008 Ω/sq for ED, 0.01 Ω/sq for EP). The different spin speeds of the nail polish solution used as CT material led to variations in domain size and metal coverage, with spin speeds ranging from 1000 rpm to 6000 rpm, affecting domain size (49 μm to 197 μm) and coverage ratio (20.5% to 25.6%).

**Figure 4:**
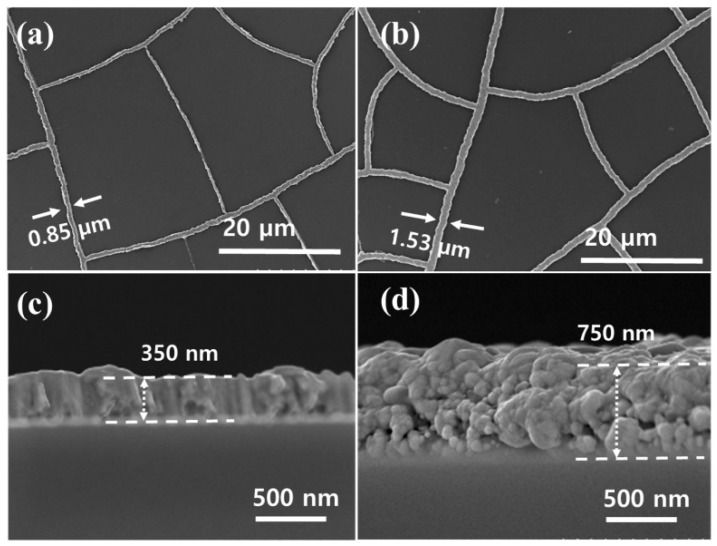
SEM images of surface and cross-section of the electrodeposited Ag mesh (onto acrylic resin crack template) grown at (**a**,**c**) 0 s and (**b**,**d**) 30 s. Reproduced with permission from ref. [70].

Similarly, Kang et al. [70] employed thermal evaporation (TE Ag) and subsequent EP (EP-TE Ag) to deposit Ag on glass and PET covered by cracked acrylic resin (Figure 4). The thermally evaporated Ag layer had a thickness of 350 nm, while the EP-TE Ag layer was 750 nm thick. The combined Ag mesh exhibited a transmittance ranging from 92% (TE) to 88% (EP-TE), with Rs of 5.7 Ω/sq and 1.01 Ω/sq, respectively. Notably, the EP Ag mesh showed higher optoelectronic performance, with a figure of merit (FoM, an important parameter used to describe the quality of TCE, see below) of 2827, compared to the individual TE-Ag mesh (756) and ITO film (311). Moreover, the EP Ag mesh demonstrated exceptional flexibility, maintaining stable Rs under both concave and convex bending tests, even with a small radius of curvature (<2 mm), making it ideal for flexible electronic applications such as solar cells and displays.

Recently, a new approach involving the ink coating and curing method to prepare Ag-based TCEs was proposed. In the work of Zarei and colleagues [39], Ag ink was drop-cast onto a PET substrate using an acrylic resin cracked template. The ink underwent a ramp-curing process that included a soft curing step to remove excess ink, followed by a hard curing step. The thickness of the resulting film, and consequently the crack patterns, was adjustable by controlling the spin-coating speed (1000–2000 rpm) and curing temperature (60–100 °C). The performance of the resulting electrodes varied across five samples, with transmittance ranging from 87.5% to 93.7% and Rs from 0.48 to 1.40 Ω/sq. After 800 bending cycles at a 4 mm radius, the Rs increased slightly (from 0.11 to 0.24 Ω/sq), but this did not significantly affect the ultimate EMI shielding effectiveness (SE) performance.

### 2.2. Copper

Cu is another widely used material in the development of TCEs due to its electrical conductivity, relatively low cost, and good mechanical properties. The thickness of Cu-based CTs typically ranges from approximately 80 nm to 3.5 μm, with variations influenced by the deposition method and the material’s interaction with the substrate. This thickness range allows for both tunable transparency and Rs, with transmittance typically ranging from 74% to 93% at 550 nm, and Rs varying from 5 to 31 Ω/sq [56,74,75]. Deposition methods for Cu include PVD techniques such as vacuum and E-beam evaporation, as well as EP and ED. These methods provide flexibility in terms of controlling the film’s thickness, crack formation, and overall performance, making Cu a versatile material for use in TCEs for flexible optoelectronic devices.

Kumar and colleagues [74] investigated the impact of wetting–drying cycles on the preparation of cracked acrylic resin templates, on both Si and PET substrates, for the successive deposition of Cu through PVD. They observed that the *Rs* of Cu films significantly decreased after 20 drying cycles, from 523 Ω/sq to 31 Ω/sq, with only a small decrease in transmittance (from 91.6% to 88.2%). This effect was due to the increase in the dimensions (length and width) of existing cracks, along with a rise in the overall crack density. To quantify the performance, they used two different *FoM*s:

- Haacke’s *FoM* [88]:

(1) $$FoM=\frac{T^{10}}{Rs}$$

measured in (Ω/sq)^−1^, where *T* is the transmittance.

- Coleman’s *FoM* [89]:

(2) $$FoM=\frac{Z_{0}}{2Rs(T^{-\frac{1}{2}}-1)}$$

dimensionless, where *Z*_0_ = 377 Ω. Higher values indicate better TCE quality. Without the drying cycles, the *FoM* values were considerably lower, but after applying the cycles, these values increased significantly, proving that the drying process enhances the quality and performance of the TCE (Table 2). These findings highlight the importance of the wetting–drying cycle in optimizing the conductivity and transparency of Cu-based TCEs.

E-beam evaporation was used by Yang et al. [78] to deposit 300 nm-thick Cu meshes on quartz substrates for EMI shielding applications. Different acrylic resin-based cracking materials (DP1–DP4) were tested, with solvent variations influencing crack morphology and metal coverage. As the composition changed from DP1 to DP4 (from hydrophilic to hydrophobic character), the metal coverage ratio dropped from 19.9% to 15.9%, leading to a rise in Rs from 6.6 Ω/sq to 11 Ω/sq, while optical transmittance ranged from 80.2% to 84.5%. Importantly, EMI SE in the 1–18 GHz range was significantly affected by crack geometry. The DP1-mesh, with wider lines and a higher duty ratio, showed the highest average SE of ~29 dB. In contrast, the DP4-mesh exhibited an average SE about 3 dB lower, demonstrating that line width and coverage are key parameters in enhancing EMI shielding performance.

**Figure 5:**
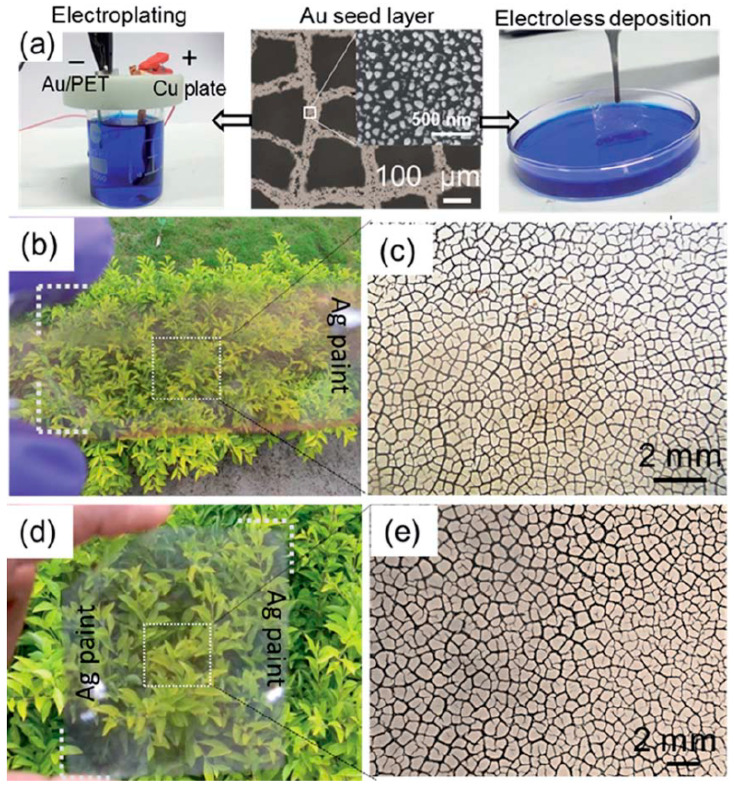
(**a**) Cu deposited onto Au seed layers using both ED and EP methods (on SiO_2_ template). Substrate images show the results of (**b**) Cu EP and (**d**) ED, with corresponding close-up microscopic views in (**c**,**e**), respectively. Reproduced with permission from ref. [73].

In the work by Kiruthika et al. [73], ED and EP were compared for fabricating Cu-based wire network electrodes on PET substrates using two types of CTs: SiO_2_ (CP-1) and acrylic emulsion (CP-2). Metal growth was initiated using Pd or Au nanoparticle seeds. The Cu film thickness reached ~100 nm for ED layer and ~300 nm for EP one, with EP mesh resulting in lower Rs due to the thicker, more conductive layer (Rs = 1.3 Ω/sq). CP-1 produced wider cracks (50–100 μm), while CP-2 resulted in narrower lines (4–8 μm) (Figure 5). Transmittance ranged between 67% and 75%, depending on the deposition method and crack geometry. Crucially, both ED- and EP-fabricated TCEs exhibited excellent flexibility and mechanical stability, maintaining consistent electrical performance even under bending radii as small as 2 mm and after multiple bending cycles, thanks to their junction-free, interconnected wire network.

Lastly, in the work of Chen and colleagues [77], thin Cu seed layer was first deposited via E-beam evaporation, followed by EP for 30–90 s, which increased the film thickness from 1.7 μm to 3.5 μm. As thickness increased, Rs dropped from 0.53 Ω/sq to 0.19 Ω/sq, while optical transmittance remained stable (from 86.4% to 85.7% at 550 nm). Using the Coleman-based FoM (Equation (2)), a near-linear improvement was observed with increasing thickness, with values ranging from 4568 to 11167. A maximum FoM of ~13,232 for 2.5 μm film was achieved with 18.6% coverage ratio, Rs of 0.18 Ω/sq, and transmittance of 85.8%. This Cu mesh outperformed other transparent conductive films such as ITO, AgNWs, graphene, CNTs, MXene, and conductive polymers analyzed in their study, with a FoM nearly two orders of magnitude higher, showcasing both high optoelectronic performance and excellent scalability for next-generation flexible and wearable devices.

### 2.3. Gold

Au has also been explored as a material for TCEs in CT architectures, primarily because of its excellent electrical conductivity, chemical stability, and resistance to oxidation. These properties render it particularly suitable for long-term and high-performance applications. Despite being less frequently used than silver due to its higher cost, Au-based meshes have demonstrated promising performance, especially in scenarios where durability and reliability are critical.

The thickness of Au CT networks typically ranges from 20 nm to 10 microns, influenced by the deposition technique and CT composition. Au meshes usually exhibit optical transmittance greater than 80% at 550 nm and Rs values ranging between 3 and 6 Ω/sq, depending on the specific structure and coverage [36,48,57].

For deposition, PVD techniques and magnetron sputtering are commonly used to create Au meshes on pre-CTs, typically formed using colloidal TiO_2_ layers, or bio-based crack materials like egg white. Specifically, colloidal TiO_2_ and egg white are widely employed as CTs for their complementary benefits: TiO_2_ enables highly uniform, low-resistance meshes with scalable precision, while egg white offers a sustainable, bio-derived alternative that promotes flexible, percolated networks through water-based, low-temperature processing.

Kiruthika and co-workers [48] employed Au as the conductive material in cracking TiO_2_-based templates, with the vacuum evaporated Au layer having a thickness of approximately 100 nm, while the TiO_2_ layer was about 10 μm thick. The crack morphology was influenced by drying temperature, where lower drying temperatures resulted in finer cracks and smaller polygon areas due to slower solvent evaporation. The substrates for this system included glass, quartz, and flexible PET. The resulting Au mesh exhibited transmittance of around 82% in the visible range, and Rs between 3 and 6 Ω/sq. The performance of the Au/PET-based TCE was further tested for adhesion and flexibility: during adhesion tests (using scotch tape), the temporary Rs change was less than 1% and observed solely during the course of the test. In flexibility tests, such as bending, crumbling, rolling and twisting, the Au/TCE showed only mild resistance changes, demonstrating its robustness.

**Figure 6:**
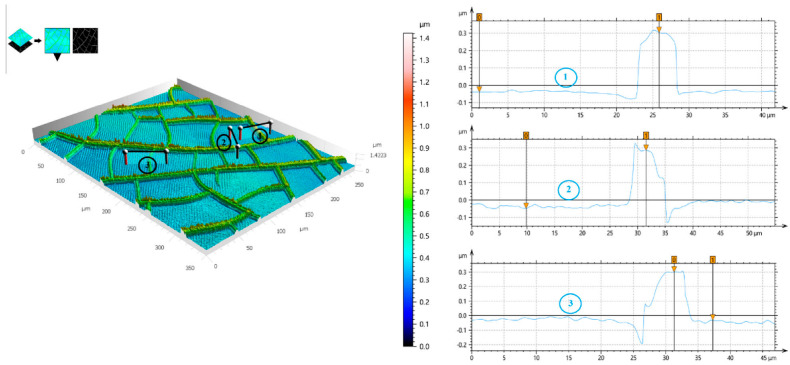
Laser scanning confocal microscope image (**left**) and probe profilometer thickness profile (**right**) of the Au mesh measured in three different test sites. Reproduced with permission from ref. [80].

In a more recent study [80], the transparent spectral range of Au metal mesh films was widened to also cover the IR region. Au metal mesh films were created on ZnS glass substrates using acrylic resin as a cracking template. Four irregularly structured metal meshes were fabricated, with varying aperture-to-line width ratios (g/w, where g is the distance between adjacent metallic lines and w is the width of the conductive metallic wires) of 16.1, 17.4, 18.0, and 19.0 and a uniform thickness of ~300 nm (Figure 6). The optical transmittance in the infrared region (2–10 μm) was found to be 57.1% to 63.2%, and the Rs ranged from 9.5 to 16.7 Ω/sq. The study also showed that the Rs decreased as the coverage ratio increased, with the lowest resistance (9.5 Ω/sq) observed for 15.33% coverage ratio. The EMI SE was excellent, exceeding 22.5 dB in the 1–18 GHz frequency range. The results confirm that the designed structural parameters of the Au mesh on ZnS substrates are effective for superior microwave shielding performance.

### 2.4. Hybrid and Other Materials

Al is a versatile and widely used metal in thin-film technology due to its excellent conductivity, low cost, and ease of deposition. It has also been extensively used in combination with other materials such as tin oxide (SnO_2_) or tungsten oxide (WO_3_) for advanced applications like smart windows or photovoltaics [62,84,85].

**Figure 7:**
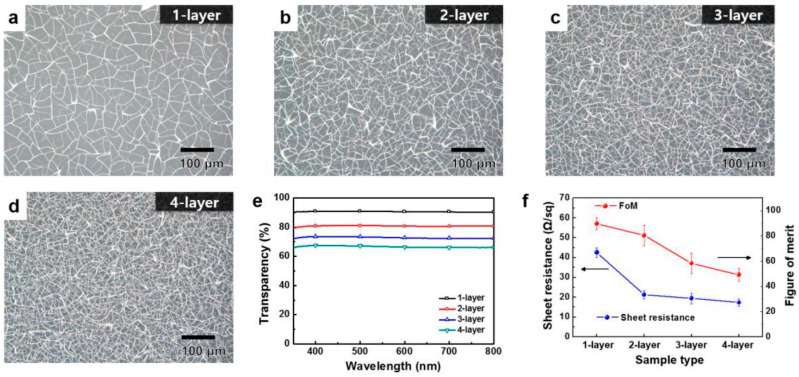
Microscope images of the Al-mesh/silica nanoparticles system: (**a**) single-, (**b**) double-, (**c**) triple-, and (**d**) quadruple-layer; (**e**) optical transparency in the visible region; (**f**) Rs and FoM of the multi-layered Al-mesh. Reproduced with permission from ref. [58].

A highly interconnected Al mesh was developed by Kim et al. [58], utilizing a self-formable CT. This template, made from colloidal silica, can be easily formed and removed, while the mesh structure can be tailored by adjusting the spin-coating speed during the application of the template solution or through the stacking of multiple metal-mesh layers. These methods allow for the precise design of the Al mesh optical transparency and Rs to meet specific requirements. For the coating of the cracking material, different spin-coating speeds were used, and the entire process was repeated four times in order to obtain multiple metallic layers (results shown in Figure 7). The double-layered metal mesh system had 80% transparency and significantly reduced Rs to around 20 Ω/sq, compared to the single-layered mesh at 40 Ω/sq. Additional layers, such as triple- and quadruple-layered mesh, decreased both transparency and FoM without significantly reducing Rs further (Figure 7f). This Al-based system was investigated for potential applications in optoelectronics and solar cells, offering a promising approach for the production of flexible TCEs.

Sn is another versatile metal that has garnered attention for its use in various electronic applications due to its favorable properties. Kiruthika’s group [59], deposited Sn using PVD method on acrylic resin as the sacrificial cracking material on a PET substrate. The resulting Sn layer had a thickness of approximately 400 nm with optical transmittance of around 80% at 550 nm, and Rs of approximately 5 Ω/sq. This system was explored for use in smart window applications, further discussed in the next section.

In recent years, hybrid materials have been investigated to combine the high conductivity of metals with enhanced mechanical, chemical, optical, or electrochromic properties, offering improved durability, cost efficiency, and tunability for advanced transparent electrode applications. Li et al. [61] successfully fabricated a WO_3_/Ag hybrid TCE for smart window applications (thus synergizing the high optical transparency of WO_3_ with the excellent electrical conductivity of Ag), using a cracked TiO_2_ template on PET substrate. After laser cutting the mesoscale lines, magnetron sputtering was used to deposit a 30 nm thick WO_3_ bottom layer, followed by the deposition of a Ag conductive layer. The optical transmittance of the hybrid material mesh was tested using grids in regular triangle, square, and hexagon shapes. The quality factors of the three hybrid TCE were found to be 1247.4 FoM (calculated using Equation (2)) for the hexagon grid (transmittance = 81%, Rs = 1.36 Ω/sq), 643.1 for the square grid (transmittance = 80.7%, Rs = 2.59 Ω/sq), and 377.2 for the triangle grid (transmittance = 80.3%, Rs = 4.31 Ω/sq), with the hexagonal grid film exhibiting the best combination of light transmission and electrical conductivity properties.

The combination of Al and SnO_2_ in hybrid electrodes also offers a cost-effective solution while maintaining high transparency. In a study by Mondal and co-workers [84], they fabricated Al-SnO_2_ hybrid electrodes with a thickness of approximately 400 nm for Al and 200 nm for SnO_2_, achieving 83% transparency at 550 nm and a Rs of 5.5 Ω/sq. These electrodes were explored for optoelectronic and photovoltaic applications.

Based on this work, in a later study, Mondal and colleagues [85] fabricated large-area Al-SnO_2_ hybrid mesh electrodes for smart window applications. To create these structures, Au and Al meshes were fabricated via thermal evaporation on a spin-coated acrylic resin as sacrificial CT. These metal meshes were subsequently functionalized with SnO_2_ coatings: the Au mesh was coated with SnO_2_ via multiple spin-coating steps and annealing, while spray deposition of a SnCl_2_ precursor at high temperatures was used for Al mesh. Al was chosen over Au mainly due to its lower cost. The resulting Al-SnO_2_ hybrid electrodes exhibited a transparency of 85% and a Rs of approximately 5 Ω/sq.

Voronin and colleagues [60] exploited the combination of Cu-Ag and Ni-Ag in hybrid micromesh electrodes. This approach preserved the excellent electrical conductivity and optical transmittance of Ag, while addressing its limitations in chemical stability and mechanical robustness. In this study, Ag layer (200 nm thick) was deposited via magnetron sputtering onto a cracked egg white template formed on PET substrates, followed by galvanic deposition of Cu (1.92 μm) or Ni (0.95 μm) to reinforce the structure. In the obtained micromesh, optical transmittance at 550 nm ranged from 82% to 88% for Cu-Ag and 78% to 87% for Ni-Ag meshes. Notably, the hybrid networks achieved ultra-low Rs: 0.06–1.52 Ω/sq for Cu-Ag and 0.7–9.3 Ω/sq for Ni-Ag, marking a substantial improvement over single-metal systems. The mechanical stability of the metal meshes on PET substrates was excellent for bending radii between 5 and 50 mm. These enhancements collectively contribute to highly efficient, durable, and cost-effective TCE for EMI shielding applications.

Other material combinations, including Au and polymers, Au and Ag, WO_3_-coated Al-SnO_2_, and vanadium-doped indium zinc oxide (IZVO, a flexible and durable alternative to ITO), have being explored for applications in a variety of devices [38,57,62,63]. The real-world applications and performance of these materials in the context of advanced devices will be further discussed in the next section.

## 3. Applications in Optoelectronics

In the following section, we will delve into the practical applications of these materials in optoelectronic devices, highlighting their performance, advantages, and potential for future technologies.

### 3.1. Electroluminescent Devices

TCEs play a vital role in the performance of electroluminescent devices, with ITO traditionally being the preferred material. However, ongoing research is increasingly focused on developing abundant, cost-effective, and high-performance transparent conductive materials to meet the growing demands of organic electronics, particularly for applications in OLED technology [90,91]. OLEDs, which have revolutionized the display and lighting industries, are renowned for their outstanding properties, such as high-luminance efficiency, full-color/large-area displays, wide viewing angles, lightness, transparency, and low power consumption [92]. Current research is aimed at creating more efficient, durable, and cost-effective transparent conductive materials, with crack-templated meshes emerging as a promising solution. There are examples demonstrating the use of crack-templated meshes technique for TCEs in OLEDs. The main parameters of device architectures and their main performances (summarized in Table 3) are discussed to highlight their advantage also in view of a possible use in flexible electronics and optoelectronics; all the devices prepared and characterized have the same active area of 2.5 × 2.5 cm^2^ to ensure comparability.

**Figure 8:**
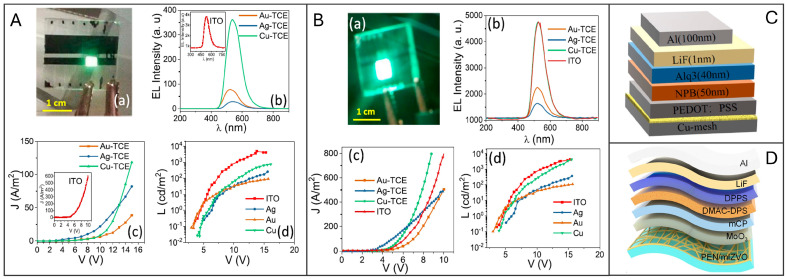
OLED characteristics with (**A**) D1 and (**B**) D2 device geometry, with Au, Ag, and Cu meshes. Subfigure A shows (a) green light emission for device D1, (b) EL spectra, (c) J–V curves, and (d) brightness–voltage characteristics compared across different metal meshes and ITO-based OLEDs under 20 mA bias. The same characterization and comparisons are presented for device D2 in subfigure B (a–d). Reproduced with permission from ref. [83]. (**C**) Device structure of OLED with Cu mesh as TCE, with the thickness indicated for each layer. Reproduced with permission from ref. [56]. (**D**) Schematic of OLED device based on IZVO mesh. Reproduced with permission from ref. [63].

In their 2020 study, Gedda et al. [83] investigated the use of metal mesh TCEs for OLEDs. It is noteworthy that, since radiative recombination is expected to occur primarily near the gridlines, it is essential to optimize the balance between key features of the metal mesh TCE such as surface roughness, Rs, and transmission. They employed PVD to deposit, upon CT film on glass, a 50 nm thick metal layer composed of Cu, Au, or Ag, followed by the lift-off process of CT to obtain TCE. The resulting mesh electrodes exhibited a high optical transmittance of 84–87% and a Rs of 7–20 Ω/sq. Moreover, the different metal used allowed for the work function (WF) tunability of anodes. Afterward, they manufactured OLED devices with two sets of different interlayers to evaluate the impact of mesh electrodes on the performance of devices (Figure 8A,B). The first type of device (D1, see Table 3) consisted of a hole injection layer (HIL) made of N,N′-Bis(3-methyl phenyl)-N,N′-diphenyl benzidine (TPD), an emitting layer (EML) of tris(8-hydroxyquinoline)aluminum (Alq3), and a hole-blocking/electron transport layer (ETL) of 4,7-Diphenyl-1,10-phenanthroline (BPhen). These layers were grown by thermal evaporation on the Cu, Au, or Ag metal mesh anode and were capped by a LiF/Al cathode. In D1, the Cu-TCE OLED showed stronger electroluminescence (EL) than Au- and Ag-based devices. The superior performance of the Cu-TCE-based OLED can be attributed to its low Rs (7 Ω/sq) and high WF of Cu (−4.7 eV), which significantly reduces the potential barrier and facilitates efficient hole injection into the EML. However, the lower performance compared to ITO-based devices indicates other interface issues that need to be addressed. The second type of device (D2) consisted of poly(3,4-ethylenedioxythiophene) polystyrene sulfonate (PEDOT:PSS) as the HTL, and Alq3 as EML with Cu mesh and LiF/Al electrodes. The deposition of PEDOT:PSS from solution to just submerge the metal wire network, provided a smoother interface than bare TCE. The brightness of the D2 configuration was enhanced to match that of the ITO OLED, with a maximum luminance (L_MAX_) of 4300 cd/m^2^ with current efficiency (CE) of 5.38 cd/A, despite having lower transmittance. These results suggest that Cu-TCEs offer similar performance to ITO-based devices, with the added advantages of cost-effectiveness and scalability.

Liu et al. [56] demonstrated a straightforward fabrication strategy for Cu network TCA utilizing a CT strategy. This method does not require complex stripping and transfer processes, is applicable to any type of metal, and can be extended to flexible substrates. Cu-based TCE were fabricated via vacuum evaporation on a PET substrate covered by a CT made by drying an acrylic based colloidal dispersion. The template was lifted-off and flushed by solvent. The optimized 120 nm-thick electrode on PET substrate exhibited transmittance of 93% at 550 nm (excluding PET substrate), Rs of 13.4 Ω/sq, root mean square roughness (RMS) as low as 4 nm and was applied as TCE in flexible OLED devices (D3). In D3, solution processed PEDOT:PSS served as HIL (similar to D2 OLED), vacuum grown N,N′-bis(1-naphthyl)-N,N′-diphenyl-1,1′-biphenyl-4,4′-diamine (NPB) as HTL, Alq_3_ as EML, and LiF/Al as cathode (Figure 8C). The D3 OLED exhibited a L_MAX_ of 1587 cd/m^2^ at 5 V and a maximum CE of about 0.75 cd/A. The device displayed uniform brightness even when curved. Additionally, the mechanical properties of the Cu-TCE were investigated: after 500 bending cycles, the network maintained Rs value with only a 5.3% change. The demonstration of a flexible OLED using these TCEs suggests their promising applicability in wearable optoelectronic devices.

A flexible electroluminescent device based on a transparent Ag network (hereafter indicated as f-NTC) was obtained and characterized by Yan and co-workers [41]. The Ag-based TCE was fabricated using magnetron sputtering on a sacrificial cracked egg white template (deposited by slot-die coating technology), on a flexible PET substrate. A fractal crackle feature formed by a thermal annealing process, where the block and line width can be modulated by a repeated wet−drying process. The electrode exhibited a transmittance of 88.1% and a Rs of 9 Ω/sq, which is comparable to widely used flexible PET/ITO or PEN/ITO. In addition, the TCE demonstrated exceptional mechanical stability, with the Rs remaining virtually unchanged even after 6000 s of bending tests. A flexible device based on f-NTCs was developed using screen printing method to deposit doped ZnS powder as EML, BaSO_4_ as a dielectric layer, and Ag paste as a cathode. The large area device (5 × 10 cm^2^) showed tunable color EL by adjusting doped elements in ZnS (Cu for blue and Cu-Mn for red light). The devices were fabricated with f-NTCs with Rs ranging between 35, 9, and 5 Ω/sq (changes in Rs of f-NTCs is achieved through wet−drying process and cycles: As the Rs decreased, luminescence intensity increased, leading to devices outperforming commercial PET/ITO-based EL panels. The intensity was uniform across the device area, and the f-NTC-based devices exhibited impressive long-term stability, maintaining 99.7% of their initial brightness (from 819 lx to 817 lx) after 35 days, compared to a decrease in intensity for the commercial device tested as control (from 380 lx to 372 lx).

Although indium remains expensive and relatively scarce, an alternative to ITO that offers flexibility and improved mechanical durability is provided by vanadium-doped indium zinc oxide IZVO. Nirmal and colleagues [63] have developed a mesh IZVO electrode (mIZVO) characterized by ultra-high transparency, superior conductivity, and exceptional mechanical durability. The incorporation of transparent conducting oxides is key to achieving its remarkable transparency. Fabrication of this electrode employs a straightforward, self-cracking, template-assisted magnetron sputtering technique on layers of sacrificial egg white that spontaneously crack under ambient conditions. The optical and electrical properties of the mIZVO electrode are precisely controlled through vanadium doping using the co-sputtering method. This approach allows for independent tuning of the WF and conductivity while maintaining optical transmittance. The effectiveness of the fabricated electrodes was tested by integrating them into OLED architectures, with performance comparisons made against IZO and ITO-based control devices. The mIZVO, with a thickness of 150 nm on a polyethylene naphthalate (PEN) flexible substrate, exhibited a transmittance of 97% and a Rs of 21.24 Ω/sq. The Haacke’s FoM [88], calculated to evaluate the trade-off between transmittance and Rs of the IZVO electrode, was 3.61 (Ω/sq)^−1^, that is higher than previously reported values. Notably, this material demonstrated effective performance in both OLED and OSC (see below) applications. The mIZVO-based OLED device structure (D4) includes a 2 nm MoO_3_ layer as HIL, a 40 nm N, N-dicarbazolyl-3,5-benzene as HTL, a 28 nm DMAC-DPS layer as EML, a 75 nm DPPS layer as ETL, a 0.8 nm LiF, and a 150 nm Al as cathode (Figure 8D). The device exhibited stable EL at 485 nm with a turn-on voltage of 3 V, and L_MAX_ slightly lower than expected due to incomplete electrode coverage. The current injection from mIZVO (WF = 5.16 eV) was more effective than from IZO (4.71 eV), IZVO (5.05 eV) and ITO (4.8 eV) based anodes, thanks to a reduced injection barrier. The corresponding external quantum efficiency (EQE) of 18.06% was higher than in IZO (14.22%) and ITO (13.29%)-based OLEDs due to better energy level alignment and high transparency. The device also showed good stability under bending, highlighting its potential for flexible applications.

The TCE approach was extended to flexible 3D electronic devices by Huang and colleagues [38]. They used hybrid bilayered Au/Ag metal grid electrodes embedded in colorless shape memory polyimide as substrates for the fabrication of flexible shape-memory-3D OLEDs. The Au/Ag hybrid electrode was fabricated using thermal evaporation sequentially, with a total thickness of 120–140 nm (consisting of 90–105 nm of Ag and 30–35 nm of Au) on self-forming CT of a commercial aqueous crackle paint, with a crack width of 2.3 µm. The CT was then cleaned up by ultrasonic cleaning in ethanol and peeling while the hybrid metal grid remains on the glass substrate. The formed TCE on glass showed a transmittance of 85% at 550 nm and a sheet resistance of 5.2 Ω/sq. The metal mesh was then embedded into a polyamic acid solution, cast on the surface, and transferred to colorless shape memory polyimide (CSMPI) substrate. CSMPI featured high transparency and thermal stability. The resulting flexible CSMPI TCE anode was incorporated into a white-emitting polymer OLED (D5) with the architecture: TCE/CSMPI/MoO_3_ (2 nm)/PEDOT:PSS (30 nm)/0.5 wt% MEH-PPV:PFO (80 nm)/Cs_2_CO_3_ (2 nm)/Al (150 nm). The resulting device switched on at 5.2 V reaching a LE of 4.3 cd/A, comparable to reference devices based on ITO anode. Notably, the flexible substrate enables transformation from 2D to diverse 3D geometries due to the shape memory properties of CSMPI. These 3D devices could recover their original planar form within seconds when heated.

### 3.2. Solar Cells

TCEs are obvious essential components also for solar cells, to provide both light transmission and charge collection. In fact, in organic and perovskite solar cells (OSCs and PSCs, respectively) there is a growing demand for TCEs able to combine high optical transparency and low electrical resistance to mechanical flexibility, scalable and low-cost fabrication flexible, wearable, and lightweight applications [93]. There has been much effort towards alternative electrodes, as reported in the literature. Graphene [94], carbon nanotubes [95] and AgNWs [96] have been proposed as alternatives to ITO and, similarly to OLED case, metal grids offer a further attractive alternative to the above TCEs [97].

Table 4 displays the main characteristics of solar cells based on CT-TCEs, including the active material, the mesh material used for the electrode, and the corresponding photovoltaic parameters.

**Figure 9:**
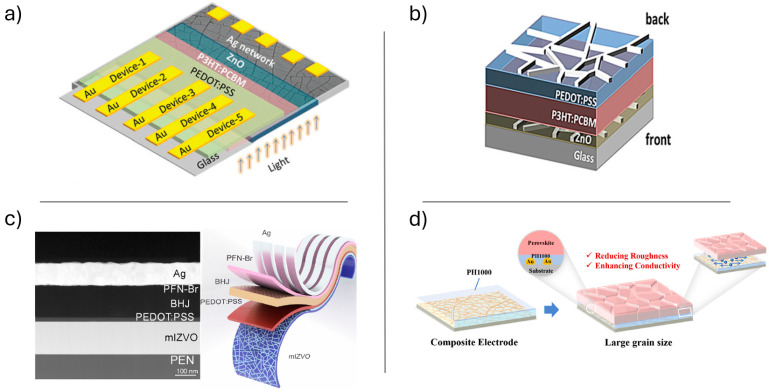
(**a**) Schematic illustration of the inverted P3HT-PCBM solar cell with Ag network TCE. Reproduced with permission from ref. [66]. (**b**) Semitransparent polymer solar cell with an Ag/Ag network as front and back electrodes. Reproduced with permission from ref. [81]. (**c**) Cross-sectional TEM image of the fabricated OSC and schematic of the OSC with IZVO as a flexible electrode. Reproduced with permission from ref. [63]. (**d**) Schematic illustration for the incorporation of PH1000 into the Au grid. Reproduced with permission from ref. [57].

For instance, a 55 nm-thick Ag-based TCE was fabricated via vacuum evaporation using an acrylic colloidal suspension as a CT on a glass substrate by Thelakkat, Kulkarni, and co-workers [66]. This electrode demonstrated good optoelectronic properties, including 86% transmittance at 550 nm and a Rs of approximately 10 Ω/sq, making it suitable for application in OSCs. Five bottom-illuminated inverted photovoltaic cells based on poly-3-hexylthiophene (P3HT) and phenyl-C61-butyric acid methyl ester (PCBM) bulk-heterojunction were fabricated using these Ag network TCEs (Figure 9a). To prevent short circuits and improve performance, CT-TCE was firstly covered by a ZnO layer with optimized thickness. A 135 nm thick ZnO layer provided full coverage of the 55 nm thick Ag network, enabling consistent performance with an average power conversion efficiency (PCE) of 2.14%, which is very much comparable to the 2.27% achieved in ITO-based devices. Though the Ag network devices showed slightly reduced EQE in the visible range, they outperformed ITO in the UV region due to superior UV transmittance. These findings highlight the viability of crack-templated Ag networks as an effective ITO alternative for OSCs, especially with tunable parameters to optimize metal network properties and compatibility with different solar cell architectures.

Notably, the application of CT-TCE can also be extended to the fabrication of the top electrode. In fact, the same group investigated in another study [81] the feasibility of utilizing CT metal networks (Ag or Au) as both front and back electrodes in ITO-free semitransparent OSCs, focusing on their integration without damaging the underlying organic layers. The authors successfully adapted the CT method, which involves forming a sacrificial template from acrylic resin nanoparticles that naturally crack during drying, to fabricate transparent metal networks directly atop soluble layers. Metals were deposited via thermal evaporation, resulting in uniform networks with metal thicknesses ranging from 20 to 60 nm and wire widths under 5 μm. After deposition, the CT was gently removed without compromising the photoactive layer. The resulting electrodes exhibited high optical transmittance (~80% across the visible spectrum) and low Rs (<5 Ω/sq). They subsequently fabricated a solar cell with this structure: glass/ZnO:Ag network/P3HT:PCBM/PEDOT:PSS/Ag network (Figure 9b). The study demonstrated an ITO-free semitransparent solar cell with a PCE of 1.8%. In the Ag/Ag network configuration, EQE values are lower between 300 and 400 nm under front illumination due to optical losses from the glass/Ag layers. However, between 400 and 650 nm, the EQE values are higher in front illumination compared to back illumination, indicating better performance in the visible spectrum when illuminated from the front. This approach provides a versatile method for fabricating transparent metal electrodes on organic layers, allowing for the use of various material combinations and metals to create different types of optoelectronic devices.

Besides the use in OLEDs discussed above, Nirmal and co-workers applied mIZVO- based TCE strategy also to OSCs [63]. Flexible OSCs with a PEN/mIZVO/PEDOT:PSS/photoactive layer/PFN-Br/Ag structure were successfully fabricated, using PM6:Y6:PC71BM as the photoactive material (Figure 9c). Under 1 sun illumination, the control device with an ITO electrode achieved a PCE of 13.17%, while the best-performing device with an mIZVO electrode achieved a PCE of 14.38%, showing enhanced performance due to the ultra-transparency and high conductivity of the mIZVO layer, together with a favorable WF alignment between IZVO and the active layer. The EQE spectra confirmed strong photoresponse in the 400–800 nm range, highlighting the potential of IZVO electrodes for efficient OSC fabrication. The possible use in both OLED and OSC technology underlines the versatility of this approach in flexible optoelectronics.

Very recently, a micro-nano fractal Au grid was fabricated using magnetron sputtering and egg white, or water-based crack glue as CT, and used as CT-TCE in PSCs [57]. The metal mesh, with a thickness of approximately 100 nm, was deposited on glass or PEN substrates, achieving about 84% transmittance at 550 nm. To further optimize conductivity and reduce roughness, high conductive PEDOT:PSS (PH1000) film was deposited by spin coating to create Au grids/PEDOT:PSS composite electrodes, improving the performance of the TCEs. The optical transmittance of the Au grids/PEDOT:PSS composite electrode exhibits minimal reduction, while the Rs significantly reduced from 5.2 to 4.18 Ω/sq with respect to bare Au grid, with a corresponding FoM as high as 566. The PSC was fabricated with the structure: Au-PEDOT:PSS/poly[bis(4-phenyl)(2,4,6-trimethylphenyl)amine] (PTAA)/perovskite/PCBM/ bathocuproine (BCP)/Ag (Figure 9d). The device achieved a PCE of 19.17%, that is very close to ITO-based PSCs performance (20.02%). The EQE showed a slight increase in the 350–550 nm range for the composite electrode, but it was lower than ITO in the 550–800 nm range, which is consistent with the transmittance results. The composite electrode-based PSCs displayed a good stability, with less than 10% efficiency decay after 4500 h, while ITO-based devices lost 50% of their initial PCE after 4000 h. This performance is comparable to traditional transparent conductive oxide devices, while also providing benefits such as flexibility, lower cost, and compatibility with large-scale manufacturing processes.

### 3.3. Smart Windows

Smart windows represent a promising solution for energy-efficient buildings by dynamically regulating light and heat transmission in response to environmental stimuli [98]. Unlike traditional passive methods, such as reflective coatings, thermal insulation, or solar protection films, that offer fixed optical properties and can be counterproductive in colder seasons, smart windows adapt to changing weather conditions, making them suitable for year-round use [99]. Technologies based on thermochromic, thermotropic and electrochromic materials are especially attractive, as they can modulate transparency with temperature or voltage [100,101,102]. Ensuring transparency, a critical component in the manufacture of smart windows, is the obvious use of transparent electrodes. ITO film is the conventional choice; however, alternative low-cost and flexible TCEs are being explored, for example involving the use of metal meshes fabricated through CT methods [103]. This technique offers a scalable, cost-effective route to produce transparent heaters using earth-abundant metals, and can be integrated with thermosensitive hydrogels, possibly becoming a viable alternative to ITO for next-generation smart window technologies.

**Figure 10:**
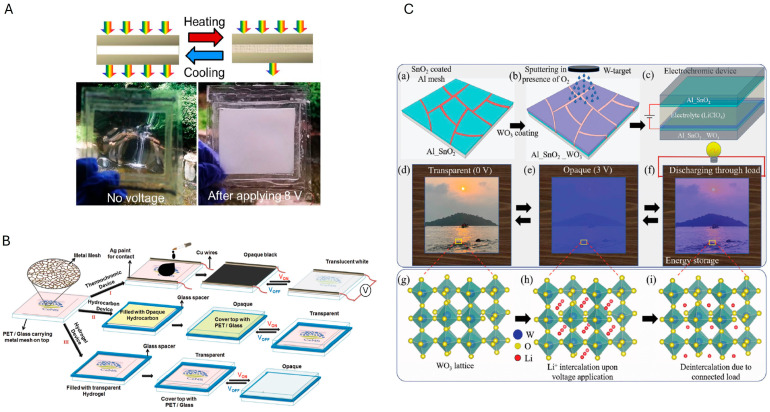
(**A**) Hydrogel device on Sn wire mesh before (left) and after (right) joule heating. Reproduced with permission from ref. [59]. (**B**) Schematic picture of the fabrication steps for (I) thermochromic device, and thermotropic devices based on (II) hydrocarbon and (III) hydrogel layer. Reproduced with permission from ref. [75]. (**C**) Fabrication of a hybrid electrode by coating SnO_2_ over an Al mesh (Al/SnO_2_, (a)), followed by the deposition of WO_3_ on the hybrid electrode (b), thus sandwiching an electrolyte between the Al/SnO_2_/WO_3_ and Al/SnO_2_ electrodes (c). In its transparent state, the window appears clear (d,g), but when voltage is applied, it transitions to an opaque state due to Li^+^ intercalation in the WO_3_ lattice (e,h). It returns to transparency by discharging through an external load (f), which depletes Li^+^ from the WO_3_ lattice (i). Reproduced with permission from ref. [62].

An example of the implementation of CT-TCE in smart window technology is presented by Kiruthika et al. [59]. In their study, a Sn metal mesh was fabricated using PVD onto a PET substrate, with an acrylic resin used to create the CT. The resulting metal network, with a wire thickness of approximately 400 nm, exhibited a high optical transmittance of ~80% at 550 nm and a low Rs of 5 Ω/sq. This combination of transparency and conductivity makes it highly suitable as a Joule heater in thermochromic smart window devices. A prototype was created by integrating the Sn mesh with a hydrogel layer made from hydroxypropyl methyl cellulose (HPMC), NaCl, and water. At lower temperatures, the hydrogel was expected to remain transparent. However, as the temperature increased upon applying a voltage to the TCE, the induced molecular aggregation caused it to turn opaque and scatter light, thereby reducing solar transmission (Figure 10A). To prevent drying, the hydrogel was sealed between the Sn wire network and PET with PDMS spacers. With a thickness of around 1 mm, the hydrogel transitioned from transparent to opaque when heated above its lower critical solution temperature (LCST) of approximately 40 °C, achieving a paper-white state with less than 1% transmittance. This transition occurred in just a few seconds with a low input power of about 0.2 W/cm^2^. In its opaque state, the device also blocked infrared radiation, a feature that could be useful to reduce temperature rise.

Interestingly, Cu electrodes can also be used for smart windows, where Cu (200 nm thick) was deposited by PVD on a cracked acrylic emulsion template on a PET substrate [75]. The structure achieved a transmittance of around 74% at 550 nm and a Rs of approximately 5 Ω/sq. Subsequently, three different thermochromic devices were fabricated for smart window applications, each using affordable and non-toxic materials (Figure 10B). Device I used a commercial thermochromic pigment with a transition temperature of ~48 °C. The pigment was dispersed in transparent acrylic paint and spin-coated over the laminated Cu mesh. Device II embedded a commonly available hydrocarbon, with transition from opaque to transparent around ~40 °C. The hydrocarbon was placed between a laminated Cu mesh electrode and a plain glass substrate, with a glass spacer and silicone adhesive securing the layers. Device III used a hydrogel made from HPMC, NaCl, and water. The hydrogel exhibited a transition from transparent to opaque at ~40 °C. Similarly to device II, it was placed between a laminated Cu mesh electrode and a glass/PET layer, with a glass spacer and silicone adhesive used to bond the layers. Device I (thermochromic pigment) was pitch black in the V_OFF_ state, and when 3.5 V was applied, it heated to over 50 °C, turning from black to translucent white with a transmittance increase from ~5% to 60%. The response time was found to be 12 s, and recovery is 13 s, with fast transitions at higher voltages. Device II (hydrocarbon) transitioned from opaque to transparent at 5 V, changing from an opaque solid to a transparent liquid above 40 °C. It showed a 70% difference in transmittance and consistent haze (65–70%) across wavelengths. Response time resulted being a few minutes, decreasing with voltage, while recovery took longer. The device was stable but not optimal for low temperatures, as it remained opaque at lower temperatures. Lastly, the hydrogel device (III) also transitioned from transparent to opaque when 5 V was applied, above 40 °C. The device showed a 79% difference in transmittance at 550 nm between V_OFF_ and V_ON_ states, with significant changes (50–70%) even in the IR region. The response time was 4 min at 4.5 V, dropping to 1 min at 7 V, with shorter recovery times than the hydrocarbon device. It showed excellent stability and is suitable for low-temperature tropical climates, remaining transparent up to 40 °C.

A smart window prototype was fabricated using an Al/SnO_2_/WO_3_ TCE with a hybrid electrode structure, designed for electrochromic applications [62]. The fabrication process involved thermal evaporation for the Al layer, spray pyrolysis for SnO_2_, and reactive ion sputtering for WO_3_, with a crackle precursor used to create the mesh pattern. The Al layer, 300 nm thick, was deposited on glass, achieving 81% transmittance at 550 nm and a Rs of about 5 Ω/sq. The smart window device operated with Al/SnO_2_/WO_3_ as the working electrode and Al/SnO_2_ as the counter electrode, with a 1 M lithium perchlorate (LiClO_4_) solution in propylene carbonate as the electrolyte. This device worked through Li^+^ intercalation into the WO_3_ lattice upon voltage application, causing a reversible transition between transparent and opaque states (Figure 10C). The electrochromic film on the hybrid electrode displayed excellent performance, with a coloration efficiency (a parameter which describes the ability of a material to modify its optical properties, such as color or transmittance, in response to an applied electrical stimulus) of ≈47 cm^2^/C and fast response and recovery times of 11 and 5 s, respectively, which is twice as fast as ITO-based control devices. The film also showed outstanding cyclic stability, retaining 95% of its performance after 500 cycles and up to 2000 cycles with the addition of a quasi-solid electrolyte (LiClO_4_ in PEO) in a two-terminal device. With its low-cost materials, this hybrid electrode offered a promising cost-effective alternative to ITO for high-performance, stable, and fast-switching electrochromic smart windows.

In addition, Ganesha and colleagues [86] fabricated an electrochromic-based smart window with energy storage capability by depositing a conductive tungsten oxide (*σ*WO_3_) film on a tungsten mesh (W-mesh) using reactive sputtering in the presence of oxygen and argon. The hybrid *σ*WO_3_/W-mesh electrodes were created by depositing the 150 nm thick *σ*WO_3_ film on a pre-fabricated 260 nm thick W-mesh electrode, which had been prepared using a cracking colloidal precursor on glass. Additionally, sub-stoichiometric WO_3-x_ films were deposited on these hybrid electrodes via reactive magnetron sputtering. Results show that the transmittance of the films ranged from 17% to 74% at 550 nm, with Rs varying between 3.2 and 19.3 kΩ/sq, depending on the argon to oxygen ratio used during the deposition process. The electrochemical studies revealed that WO_3-x_/*σ*WO_3_/W-mesh electrodes exhibited larger areas under cyclic voltammetry curves compared to ITO-based electrodes, indicating higher areal capacitance. The coloration efficiency of these hybrid electrodes was 51.23 cm^2^/C at 550 nm and 76.02 cm^2^/C at 632 nm, among the highest for sputtered films, and the response and recovery times for the WO_3-x_/*σ*WO_3_/W-mesh electrodes were 29 and 14 s, respectively, showcasing their fast-switching capabilities. The electrodes also showed excellent stability, maintaining 71% and 70% transmittance retention at 550 nm and 632 nm, respectively, after 2000 switching cycles.

### 3.4. Other Devices

The CT approach used for TCE fabrication has demonstrated broad versatility and has been successfully applied also for other applications, specifically touch screen devices and wearable sensors.

**Figure 11:**
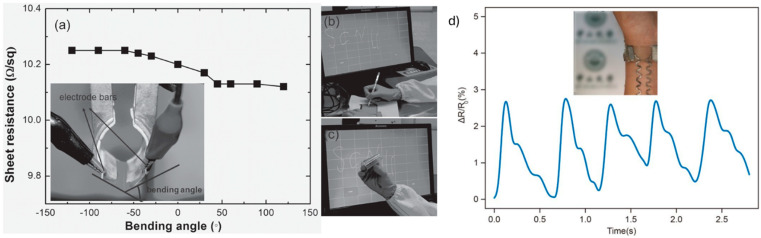
(**a**) Rs of Ag network as a function of the bending angle. (**b**,**c**) A functional prototype of a flexible touch-screen display fabricated with Ag network. Reproduced with permission from ref. [65]. (**d**) Stretchable and transparent electrode detecting pulse wrist gesture. Reproduced with permission from ref. [69].

Han et al., created an Ag-based TCE using a TiO_2_ CT followed by metal deposition through thermal evaporation and sputtering. The resulting Ag mesh, about 60 nm thick with wire widths between 1 and 2 μm, was formed on both PET and glass substrates. The electrode achieved high optical transparency over 80% at 550 nm, and low Rs, measuring 10 Ω/sq on PET and 4.2 Ω/sq on glass. Thanks to this balance of transparency and conductivity, the TCE was applied in a touch screen device prototype (Figure 11b,c). The device demonstrated optimal electro-optical performance, with a FoM (calculated using Equation (2)) ranging from 300 to 700, which is among the highest reported for such structures. Mechanical flexibility tests showed that the Ag network on PET retained stable conductivity over a wide bending range (±120°) with minimal, reversible variation in resistance, even after repeated bending cycles and touch interactions (Figure 11a). Additionally, the hydrophobic nature of the network promotes self-cleaning, preventing the accumulation of dirt and water, which could lead to a new range of potential applications [65].

The growing demand for wearable, flexible, and non-invasive sensing platforms—particularly for real-time physiological monitoring in sports and healthcare settings—has accelerated the need for mechanically robust and highly conductive transparent electrodes suitable for integration into next-generation biosensors [104]. Thus, another promising application for a metal-based crack-templated mesh was studied by Urgunde and co-workers [79] and involved the fabrication of an enzymeless glucose sensor. The metal, Au in this case, was deposited via PVD on a crackle paint surface, on glass and PET substrates, reaching approximately 60% of transmittance and Rs of 4 Ω/sq. Nickel butylthiolate (Ni-C4SH) was employed as a coating for its electrocatalyst properties. The electrochemical performance of Ni-C4SH-functionalized Au mesh electrode was investigated for glucose sensing applications and compared to Ni-C4SH/Au film electrode. The Au mesh electrode demonstrated a lower oxidation potential compared to the Au film, offering better glucose detection at reduced voltages (0.45 V for Au mesh and 0.55 V for Au film). The glucose detection range for the Au mesh electrode was broader (0.5–11 mM) compared to Au film (0.03–8 mM). The Au mesh also demonstrated higher sensitivity and lower limits of detection, making it more suitable for glucose sensing, particularly in blood samples. Furthermore, it exhibited good stability, reproducibility, and minimal interference from other molecules. When tested with a diluted blood sample, the Au mesh electrode showed significant current response, highlighting its potential for real-time glucose monitoring.

In view of fabricating stretchable strain sensors, AgNWs blade-coated onto fluoropolymer layer (cracked due to tensile strain) on a PDMS substrate, were developed [69]. The mesh showed a transmittance of approximately 89.66% and a Rs of 9.8 Ω/sq. The strain-resistance characteristics of the device showed a high gauge factor (defined as the ratio of the relative change in electrical resistance to the mechanical strain applied to the material) of 1048 at 80–100% strain and 500 at 125% (maximum tensile strain). When used as a strain sensor, the device effectively monitored human activities like pulse, hand gestures, and joint movement, providing reliable, real-time data (Figure 11d). It has also shown high mechanical durability with no fatigue after 1000 strain/release cycles, confirming its suitability for wearable electronics and human health monitoring applications.

## 4. Conclusions and Future Perspectives

Crack-templated fabrication techniques have demonstrated remarkable potential as a low-cost, scalable, and highly effective strategy for developing TCEs, especially as alternatives to brittle and expensive materials like ITO. Across the various studies reviewed, a recurring theme is the fine control of crack morphology (e.g., thickness and width), which directly influences the optical transmittance, Rs, conductivity, and mechanical response of the final electrode. By leveraging naturally forming or engineered cracks in templates such as dried polymer films or thin colloid layers, researchers have been able to generate well-defined metallic mesh networks with precise geometry and tunable properties. This precise control is crucial for balancing optical transparency with electrical conductivity.

The performance of crack-templated TCEs arises from the synergistic interaction between the cracking material, the mesh material, and the chosen deposition technique. The cracking material dictates the morphology of the template—controlling features like crack width, density, and pattern orientation—which in turn define the trade-off between optical transparency and electrical conductivity. For example, stiffer ceramic templates usually yield more regular cracks suited for high-resolution metal meshes, while soft polymeric layers allow for flexible or stretchable architectures [71,105]. The mesh material further tunes performance: highly conductive metals like Ag enable low sheet resistance, but their stability depends on both the underlying template and the deposition method [106]. Deposition technique plays a crucial role in determining the quality of metal infiltration into the crack network—evaporation ensures conformal coating and minimal overfilling, whereas electrodeposition enables selective filling but may suffer from non-uniform growth if the template or surface energy is not optimized [107]. When these parameters are effectively matched, their combined characteristics enable precise control over the resulting electrode’s structure and function. This synergy allows for the fine-tuning of electrical conductivity, optical transparency, mechanical flexibility, and chemical stability, making it possible to optimize TCE performance for the requirements of a wide range of applications.

Beyond electrical and optical performance, uniformity and reproducibility have emerged as critical challenges and priorities. Advances in crack control, for example through surface treatments, are enabling better repeatability in mesh formation. Durability and environmental stability are also essential for practical deployment. Several studies address this by embedding conductive materials within protective crack architectures or additional materials, reducing oxidation risk or mechanical fatigue, which has resulted in devices withstanding over several thousands of strain cycles without performance degradation.

The application of CT-TCE to a variety of device technologies, including OLEDs, OSCs, PSCs, smart windows, and sensors, has been extensively reviewed. These CT-TCEs demonstrate performance largely comparable to that of traditional ITO-based devices. Specifically, they offer similar electrical conductivity and optical transparency, which are crucial for the efficient functioning of these devices. Furthermore, the integration of CT-TCEs into these technologies promises advances in device flexibility, cost-effectiveness, and improved environmental sustainability, thereby underscoring their potential as a viable alternative to ITO.

From an industrial perspective, crack-templating is particularly promising due to its compatibility with solution-processable and low-temperature techniques. These methods are not only cost-effective but also compatible with flexible substrates such as PDMS, PET, and PEN, making them suitable for large-area manufacturing. This opens doors to hybrid integration strategies, where crack-based metal meshes are combined with nanomaterials—such as graphene, metal nanowires, and conductive oxides—to tailor device properties for specific applications, from flexible displays and solar cells to wearable strain sensors and human–machine interfaces. In terms of sustainability, their compatibility with flexible and biodegradable substrates, which could be cellulose or eco-friendly polymers, enhances the potential for green electronics and disposable sensor platforms. The structural efficiency of crack-defined meshes also allows for minimal use of metal, reducing material consumption without compromising performance. In terms of scalability, devices fabricated with crack-templated electrodes currently range from small-area formats (2.5 × 2.5 cm^2^) to intermediate sizes such as 15 × 15 cm^2^ [86], 15 × 20 cm^2^ [40], and up to A4 dimensions (21 × 29.7 cm^2^) [73], demonstrating the feasibility of large-area implementation. This progression highlights the potential of CT-based methods to support practical, industrial-scale applications.

Looking forward, future development in this field should focus on optimizing crack templating mechanisms for deterministic patterning, enhancing interfacial adhesion between mesh and substrate for improved mechanical robustness, and expanding the material palette to include biocompatible or self-healing systems. Additionally, integrating machine learning and advanced simulation tools for predictive control of crack behavior and performance metrics could significantly accelerate development. Ultimately, crack-templated TCEs represent a versatile and evolving platform capable of enabling the next generation of flexible, stretchable, and transparent electronics, bridging the gap between high-performance lab-scale devices and scalable industrial applications.

## Figures and Tables

**Table 1:** Summary of crack-template works in the literature, highlighting key parameters such as mesh material, metal deposition technique, crack template material, thickness, substrate, transmittance, resistance, and their respective applications.

Mesh Material	Metal Deposition Technique	Crack Template Material	Thicknesses	Substrate	Transmittance	Resistance	Figure of Merit (FoM, Coleman)	Applications	Ref.
Ag	Physical vapor deposition	Acrylic resin (CP1), SiO_2_ (CP2)	80 nm (Ag) 0.5–30 μm (CP1) 24–150 μm (CP2)	Glass tube; convex lens; round-bottom flask	86% (CP1) 70% (CP2)	6 Ω/sq (CP1) 2 Ω/sq (CP2)	401 (CP1) 482 (CP2)	Transparent heaters, curved surfaces	[64]
Ag	Thermal evaporation; Sputtering	TiO_2_	~60 nm (Ag) 1–2 μm (wire width)	PET, glass	>80% (550 nm)	10 Ω/sq (PET) 4.2 Ω/sq (glass)	300–900	Touch screen device	[65]
Ag	Vacuum evaporation	Acrylic colloidal suspension	55 nm (Ag)	Glass	86% at 550 nm	~10 Ohm/sq	n.a.	OSC	[66]
Ag	Drop casting (AgNWs)	High-stress thin silicon nitride (Si_3_N_4_)	40 nm (AgNW diameter) 1.65–2.05 μm (Si_3_N_4_ film)	Silicon wafer	85%	0.77 Ω/sq	n.a.	Touch screens	[34]
Ag	Electroless deposition; Electroplating	Nail polish/egg white	100 nm (Ag electroless deposition) 2.5 μm (Ag electroplated)	Glass	80–82% (pre- and post-electroplating)	0.008 (pre)–0.01 (post) Ω/sq	Up to 1000 (pre)–208.000 (post)	Transparent conductor for, e.g., OLEDs, thin film solar cells, and EMI shielding	[67]
Ag	Electroplating	Egg white/ Nail polish + polyimide (PI)	100–400 nm (Ag)	Silicon	~85%	1.5 Ω/sq	700–1400	Photovoltaics	[37]
Ag	Sputtering	Acrylic resin	100 nm (Ag)	PET	~86%	~6 Ω/sq	408	Thermochromic devices	[28]
Ag	Magnetron sputtering	Egg white 1 mL/L (template A) or 3 mL/L (template B)	200–600 nm (Ag)	PET	84–91% (550 nm)	1.6–21 Ω/sq	n.a.	Transparent heaters (e.g., anti-fogging and anti-icing coatings)	[68]
Ag(NWs)	Blade coating	Fluoropolymer	10 μm (wire width)	PDMS	~90% (550 nm)	9.8 Ω/sq	360	Strain sensors	[69]
Ag	Thermal evaporation; Electroplating	Acrylic resin	-Thermally evaporated Ag (TE Ag): 350 nm -EP Ag (EP TE Ag): 750 nm	Glass; PET	TE: 92% EP TE: 88%	5.7 Ω/sq 1.01 Ω/sq	2827	Flexible transparent electrodes	[70]
Ag	Thermal evaporation	ZnO + PVP	200 nm (Ag) 6 μm (crack layer)	Quartz; sapphire; glass	~83% (550 nm) (quartz > sapphire and glass)	Quartz = 13.55 Ω/sq; sapphire = 10.45 Ω/sq; glass = 13.65 Ω/sq	n.a.	Encryption, solar cells, touch panels, light-emitting diodes, transparent heaters	[71]
Ag	Magnetron sputtering	Egg white	2.5 μm (Ag) 4 nm (PMMA spacer in sandwich structure)	PET	~89%	0.28–1.59 Ω/sq	1303–5495	EMI shielding	[72]
Ag	Magnetron sputtering	Egg white	~130 μm (cracks) 3–6–10 μm (Ag network, after 1 and 2 wet/dry cycles) 15 μm (Ag-Re micro-nanosheets)	PET	~88.1%	~9 Ω/sq	n.a.	Flexible display technologies, lighting, sensing	[41]
Ag	Ag ink coating and curing	Acrylic emulsion (CA-600)	~1.8 μm (Ag) <5 μm widths, 5 μm depths (cracks)	PET	91–93%	0.54–1.4 Ω/sq	4070–7500	Transparent electrodes and EMI shielding	[39]
Ag	Magnetron sputtering	Egg white	50 μm (egg white) 100–300–600 nm (Ag) 6 μm (wire width)	PET	>80% (500 nm)	~1.84 Ω/sq	n.a.	Electronic and optoelectronic, EMI shielding	[42]
Cu	Electroless deposition; Electroplating	SiO_2_ (CP1), Acrylic emulsion (CP2) + Pd/Au	300 nm (EP Cu) 100 nm (ELD Cu) 50–100 μm (CP1 width) 4–8 μm (CP2 width)	PET	67–75%	3.8 Ω/sq (ELD + Pd) 0.3 Ω/sq (EP + Au) 1.3 Ω/sq (ELD + Au)	n.a.	Large-area applications (e.g., large panel displays), touch screens and solar cells	[73]
Cu	Physical vapor deposition	Acrylic resin	~1.7 μm (film) ~700 nm (crack width)	Si; PET	88.2%	31 Ω/sq	71–95	Touch screens, solar cells	[74]
Cu	Physical vapor deposition	Acrylic emulsion	200 nm	PET	~74% (550 nm)	~5 Ω/sq	n.a.	Smart windows	[75]
Cu	n.a.	Acrylic resin	80 nm (Cu) ~400 nm (crack width) ~15 μm (crack spacing)	Glass	~87.5%	10 Ω/sq	n.a.	Optoelectronic applications (e.g., solar cells, touch screens, transparent heaters)	[76]
Cu	Vacuum evaporation	Acrylic suspension	120 nm	PET	93% (550 nm)	13.4 Ω/sq	380	OLED	[56]
Cu	E-beam evaporation; Electroplating	Acrylic emulsion	1.7–3.5 μm (Cu)	Ni sheet	85.8% (550 nm)	0.18 Ω/sq	13.232	EMI shielding	[77]
Cu	E-beam evaporation	Acrylic resin + water (DP1) + water and ethylene glycol (DP2) + water and glycerol (DP3) + water and NMP (DP4)	300 nm (Cu) Crack width: 2–4 μm	Quartz	80–85% (550 nm)	6–11 Ω/sq	n.a.	EMI shielding	[78]
Au	Vacuum evaporation	TiO_2_	~100 nm (Au) ~10 μm (TiO_2_)	Glass; quartz; PET	~82%	3–6 Ω/sq	n.a.	Optoelectronics	[48]
Au	Physical vapor deposition	Crackle paint	n.a.	PET/glass	~60%	~4 Ω/sq	n.a.	Enzymeless glucose sensors	[79]
Au	E-beam evaporation	Acrylic resin	300 nm	ZnS	57.1% to 63.2% (2–10 μm)	9.5–16.7 Ω/sq	n.a.	EMI shielding	[80]
Au, Cu, Ag, Pd, Al, Zn	Physical vapor deposition	Acrylic resin	90–800 nm (metal wire) 1–4 μm (film)	Glass; quartz; PET	90.5% (240–3000 nm)	2.6 Ω/sq (Ag)	n.a.	Transparent, flexible heaters	[40]
Ag, Au	Thermal evaporation	Acrylic resin nanoparticles	20–60 nm (Au/Ag) < 5 μm (wire width)	PEDOT:PSS-coated glass	>80%	<5 Ω/sq	765	Polymer solar cells	[81]
Ag, Cu	Thermal evaporation and sputtering	Egg white	60–6000 nm (after electroplating)	PET	60–95%	0.03–3 Ω/sq	10.000–30.000	LED lighting, solar cells	[82]
Cu, Au, Ag	Physical vapor deposition	Crackle precursor (CP)	50 nm (metal)	Glass	>85%	~7 Ω/sq	n.a.	OLED	[83]
Al	Radio frequency (RF) magnetron sputtering	Silica nanoparticles	~800 nm (silica) 150 nm (Al)	Glass	~80% (300–800 nm)	~20 Ω/sq	40 < FoM < 90	Optoelectronics, solar cells	[58]
Sn	Physical vapor deposition	Acrylic resin	400 nm	PET	~80% (550 nm)	5 Ω/sq	n.a.	Smart windows	[59]
Au/Ag	Thermal evaporation	Crackle paint (BMG1–4)	Ag: 90−105 nm Au: 30−35 nm (Tot: 120–140 nm) Crack width: 2.3 um	Glass	85% (550 nm)	5.2 Ω/sq	428	White polymer light emitting diodes (WPLEDs)	[38]
WO_3_/Ag	Magnetron sputtering	TiO_2_	30 nm (WO_3_) 3–10 μm (crack width)	PET	81%	1.36 Ω/sq	377–1247	Smart windows	[61]
Al-SnO_2_	Thermal evaporation	Crackle precursor	~400 nm (Al) ~200 nm (SnO_2_)	Glass	~83% (550 nm)	5.5 Ω/sq	n.a.	Optoelectronics and photovoltaics	[84]
Al-SnO_2_, Au-SnO_2_	Thermal evaporation	Acrylic resin	<30 μm	Glass	85% (Al-SnO_2_) 92% (Al) 84% (Au-SnO_2_)	5 Ω/sq (Al-SnO_2_) 8–10 Ω/sq (Au-SnO_2_)	n.a.	Smart windows	[85]
Cu-Ag, Ni-Ag	Galvanic deposition (Cu, Ni), Magnetron sputtering (Ag)	Egg white	1.92 μm (Cu) 0.95 μm (Ni) 200 nm (Ag)	PET	82–88% (Cu-Ag) 78–87% (Ni-Ag) (550 nm)	0.06–1.52 Ω/sq (Cu-Ag); 0.7–9.3 Ω/sq (Ni-Ag)	292 (Ni-Ag)–1785 (Cu-Ag)	EMI shielding	[60]
Al_SnO_2__WO_3_	Thermal evaporation (Al); Spray-pyrolysis (SnO_2_); Reactive ion sputtering (WO_3_)	Crackle precursor	300 nm (Al)	Glass	~81% (550 nm)	~5 Ω/sq	n.a.	Smart windows	[62]
WO_3_	Direct current (DC) sputtering	Colloidal precursor	260 nm	Glass	17–74% (550 nm)	3.2–19.3 Ω/sq	n.a.	Smart windows	[86]
V-doped IZO (IZVO)	Magnetron sputtering	Egg yolk	150 nm (mesh)	PEN	97%	21.24 Ω/sq	3.61 (Ω/sq)^−1^ (Haacke)	OLED and OSC	[63]
Au/ Au+PH1000	Magnetron sputtering	Egg white/water-based crack glue	~100 nm	Glass; PEN	84% (550 nm)	4 Ω/sq	566	Perovskite solar cells (PSC)	[57]

n.a. = not available.

**Table 2:** Summarized table of FoM results for Cu-based mesh before and after the application of wetting-drying cycles.

Sample	Haacke FoM (Before Drying) [(Ω/sq)^−1^]	Haacke FoM (After Drying) [(Ω/sq)^−1^]	Coleman FoM (Before Drying)	Coleman FoM (After Drying)
Sample 1	7.95 × 10^−4^	9.2 × 10^−3^	8	94.7
Sample 2	9.1 × 10^−4^	6.3 × 10^−3^	9.3	71.2
Sample 3	1.7 × 10^−3^	7.1 × 10^−3^	17	86.9

**Table 3:** Summarized architecture details and devices performance of OLEDs featuring crack-templated TCEs. Performance is evaluated in terms of luminance (L), external quantum efficiency (EQE), current efficiency (CE), turn on voltage, Rs, Haacke’s FoM, when available.

OLED Device	Anode (Mesh)	Hole Injection Layer (HIL)	Hole Transport Layer (HTL)	Emitting Layer (EML)	Electron Transport Layer (ETL)	Electron Injection Layer (EIL)	Cathode	Substrate	Performance	Ref.
D1	Cu Au Ag	TPD	/	Alq_3_	BPhen	LiF	Al	Glass	L_MAX_ of 4300 cd/m^2^, CE of 5.38 cd/A, similar performance to ITO	[83]
D2	Cu	PEDOT:PSS		Alq_3_		LiF	Al	Glass	Enhanced L, similar performance to ITO-based devices	
D3	Cu	PEDOT:PSS	NPB	Alq_3_	Alq_3_	LiF	Al	PET	L_MAX_ of 1587 cd/m^2^, 500 bending cycles with minimal Rs change	[56]
D4	IZVO	MoO_3_	mCP	DMAC-DPS	DPPS	LiF	Al	PEN	EQE of 18.06%, FoM of 3.61 (Ω/sq)^−1^, high transmittance (97%)	[63]
D5	Au/Ag	MoO_3_	PEDOT:PSS	MEH-PPV:PFO	/	Cs_2_CO_3_	Al	CSMPI film	Turn-on voltage of 5.2 V, CE of 4.3 cd/A, flexible and 3D-formable substrate	[38]

TPD: N,N′-Bis(3-methylphenyl)-N,N′-diphenylbenzidine. Alq_3_: *Tris(8-hydroxyquinolinato)aluminum.* BPhen: Bathophenanthroline. PEDOT:PSS: Poly(3,4-ethylenedioxythiophene) polystyrene sulfonate. MEH-PPV: Poly[2-methoxy-5-(2-ethylhexyloxy)-1,4-phenylenevinylene]. PFO: Poly(9,9-di-n-octylfluorenyl-2,7-diyl). NPB: N,N′-Di(1-naphthyl)-N,N′-diphenyl-(1,1′-biphenyl)-4,4′-diamine. MoO_3_: *Molybdenum trioxide.* mCP: N, N-dicarbazolyl-3,5-benzene. DMAC-DPS: 10,10′-(4,4′-Sulfonylbis(4,1-phenylene))bis(9,9-dimethyl-9,10-dihydroacridine. DPPS: Diphenyl-bis(4-(pyridin-3-yl)phenyl)silane. CSMPI: colorless shape memory polyimide. PET: Polyethylene terephthalate. PEN: Polyethylene naphthalate.

**Table 4:** Summary of key performance parameters for solar cells incorporating CT-TCEs. Performance is evaluated in terms of short-circuit current density (J_sc_), open-circuit voltage (V_oc_), fill factor (FF), and power conversion efficiency (PCE).

Active Material	Mesh Material	J_SC_ (mA/cm^2^)	V_OC_ (V)	FF (%)	PCE (%)	Ref.
P3HT:PCBM	Ag	~8.3 (Ag); ~8.6 (ITO)	~0.60	~43–44	2.14 (Ag); 2.27 (ITO)	[66]
P3HT:PCBM	Ag, Au	~7.2 (Ag/Ag); ~9.4 (ITO/Ag)	~0.59–0.62	~55–60	1.80 (Ag/Ag); 2.25 (ITO/Ag); 3.10 (ITO/Ag opaque)	[81]
PM6:Y6:PC71BM	m-IZVO	27.7 (ITO); >27.7 (m-IZVO)	~0.689	~69	14.38 (m-IZVO); 13.17 (ITO)	[63]
PTAA:PVK:PCBM:BCP	Au-PH1000	21.82 (Au-PH1000); 22.02 (ITO)	1.11	78.99	19.17 (Au-PH1000); 20.02 (ITO)	[57]

## Data Availability

No new data were created or analyzed in this study. Data sharing is not applicable to this article.

## Abbreviations

The following abbreviations are used in this manuscript:

CE	Current Efficiency
CNTs	Carbon Nanotubes
CSMPI	Colorless Shape Memory Polyimide
CT	Crack Template
ED	Electroless Deposition
EIL	Electron Injection Layer
EML	Emissive Layer
EP	Electroplating
EQE	External Quantum Efficiency
ETL	Electron Transport Layer
FoM	Figure of Merit
HIL	Hole Injection Layer
HPMC	Hydroxypropyl Methyl Cellulose
HTL	Hole Transport Layer
ITO	Indium Tin Oxide
IZVO	Indium Zinc Vanadium Oxide
LCST	Lower Critical Solution Temperature
OLED	Organic Light-Emitting Diode
OSC	Organic Solar Cell
PCE	Power Conversion Efficiency
PET	Polyethylene Terephtalate
PEN	Polyethylene Naphtalate
PDMS	Polydimethylsiloxane
PVD	Physical Vapor Deposition
PSC	Perovskite Solar Cell
Rs	Sheet Resistance
TCE	Transparent Conductive Electrode
WF	Work Function
